# Catch Me If You Can! RNA Silencing-Based Improvement of Antiviral Plant Immunity

**DOI:** 10.3390/v11070673

**Published:** 2019-07-23

**Authors:** Fatima Yousif Gaffar, Aline Koch

**Affiliations:** Centre for BioSystems, Institute of Phytopathology, Land Use and Nutrition, Justus Liebig University, Heinrich-Buff-Ring 26, D-35392 Giessen, Germany

**Keywords:** RNA silencing, Host-induced gene silencing, Spray-induced gene silencing, virus control, RNA silencing-based crop protection, GMO crops

## Abstract

Viruses are obligate parasites which cause a range of severe plant diseases that affect farm productivity around the world, resulting in immense annual losses of yield. Therefore, control of viral pathogens continues to be an agronomic and scientific challenge requiring innovative and ground-breaking strategies to meet the demands of a growing world population. Over the last decade, RNA silencing has been employed to develop plants with an improved resistance to biotic stresses based on their function to provide protection from invasion by foreign nucleic acids, such as viruses. This natural phenomenon can be exploited to control agronomically relevant plant diseases. Recent evidence argues that this biotechnological method, called host-induced gene silencing, is effective against sucking insects, nematodes, and pathogenic fungi, as well as bacteria and viruses on their plant hosts. Here, we review recent studies which reveal the enormous potential that RNA-silencing strategies hold for providing an environmentally friendly mechanism to protect crop plants from viral diseases.

## 1. Introduction

### Antiviral Plant Defence Responses

Plant viruses are submicroscopic spherical, rod-shaped or filamentous particles which contain different kinds of genomes. The majority of plant virus genomes consist of single-stranded RNA (ssRNA). Nevertheless, some viruses also have double-stranded RNA (dsRNA), ssDNA or dsDNA as genomic sources [1]. The nucleic acid is encapsulated by a coat or capsid consisting of one or several types of protein molecule [2]. Virus infection of plant cells is mainly mediated by vectors (e.g., aphids, whiteflies, nematodes, plasmodiophorids or chytrids) [3,4] but can also occur through mechanical wounds as well as vertical transmission via seeds [5,6]. Viral multiplication inside the plant depends on host cellular mechanisms that support the replication of the viral genomes and cell-to-cell, as well as systemic movement of the virus via plasmodesmata and the connected phloem [7]. After invading the plant, viruses infect a small region, causing, for example, small patterns of a greenish yellow mosaic, which can spread as the virus distributes through the plant vasculature to distant plant organs or to other host plants by vector-mediated transmission [8,9]. Phytoviruses cause numerous severe diseases; thus, they have an obligate great economic impact on agricultural productivity. However, plants have evolved a highly complex, tightly regulated, and multi-layered immune system to combat viral pathogens. Specifically, the infection and replication of viruses in the host plant induce diverse defence mechanisms for combating viral infection, including (i) innate immunity, (ii) RNA silencing, (iii) translational repression, (iv) ubiquitination-mediated protein degradation, (v) nonsense-mediated decay, and (vi) non-stop and no-go decays [10,11,12,13,14,15,16]. The major defence mechanisms, innate antiviral immunity, and RNA silencing are discussed only briefly since they were reviewed extensively previously [12,13].

In plants, pattern recognition receptors located on the plant cell membrane detect the presence of pathogen- and microbe-associated molecular patterns (PAMPs/MAMPs) and activate PAMP-triggered immunity (PTI) [17,18,19,20]. While PTI is frequently sufficient to prevent pathogen colonization, some pathogens have evolved effector proteins or small RNA effectors that suppress PTI [21,22,23]. Plants, in turn, have evolved R-proteins that, following direct or indirect interaction with their cognate pathogen encoded effector, also known as avirulence (Avr) proteins, trigger effector-triggered immunity (ETI; also called R-gene-mediated resistance) [24,25,26]. Both PTI and ETI are associated with the activation of immune responses in the inoculated tissue, such as the synthesis of anti-microbial compounds, generation of reactive oxygen species, expression of defence-associated genes, including PATHOGENESIS-RELATED (PR)-1, and the accumulation of the defence-signalling hormone salicylic acid. Whereas innate immunity in plant defence against non-viral pathogens is well described in the literature, there is still a considerable gap of knowledge regarding the role of PTI against plant viruses. Moreover, the controversy on whether plant viruses are recognized as PAMP-coding pathogens is still a matter of debate; however, recent findings indicate that the detection of dsRNAs (produced as result of virus replication during infection) as viral PAMPs involves typical PTI components, and the induced immune response differs from those of the RNA-silencing pathway [27,28,29,30,31]. To overcome host immune responses, viruses counteract plant PTI via the generation of viral effectors. Viral Avr proteins comprise, for example, movement proteins, replicase proteins and coat proteins (CPs), which function invariably as virulence factors necessary for successful infection. However, viral effectors are sensed by host R-proteins that trigger a cascade of downstream signalling events that induce R-gene mediated resistance (ETI). How those defences and counter-defences orchestrate in plant-virus interactions is not in the scope of this review, and readers are referred to recent reviews [10,11,12,13,32,33].

## 2. RNA Silencing-Mediated Antiviral Plant Immunity

RNA silencing (also termed RNA interference, RNAi) is a conserved regulatory mechanism of gene expression in eukaryotic organisms which is triggered by dsRNA-provoking gene silencing by sequence-specific degradation of complementary mRNA transcripts (post-transcriptional gene silencing, PTGS) [34,35] or by inhibition of transcription (transcriptional gene silencing, TGS) [36]. RNA silencing plays a pivotal role in diverse cellular, developmental and physiological processes, regulating gene expression via small noncoding RNAs (sRNAs) [37] and is associated with protection against viral infection, the control of epigenetic modifications, regulation of genome stability, curbing of transposon movement and regulation of heterochromatin formation [36].

The ultimate trigger initiating/eliciting RNA silencing is dsRNA, which is a replication intermediate generated by viral RNA-dependent RNA polymerases (RDRs) of plant-infecting RNA viruses [38]. RNA-silencing mechanisms start with initial processing or cleavage of a precursor dsRNA into short 21–24 nucleotide (nt) small interfering (siRNA) or micro RNA (miRNA) duplexes [39] by an RNaseIII-like enzyme called Dicer (DCL) [40,41]. Double-stranded siRNAs are incorporated into an RNA-induced silencing complex (RISC) containing an Argonaute (AGO) protein that has a sRNA-binding domain and an endonucleolytic activity for cleavage of target RNAs [42]. The activated RISC subsequently unwinds siRNAs, thereby generating a sense (passenger) and an antisense (guide) strand in an ATP-dependent reaction. While the sense strand is degraded, the RISC containing the antisense strand subsequently targets a complementary mRNA transcript via base pairing interaction, degrades the mRNA and thereby inhibits protein biosynthesis [43,44,45].

**Figure 1:**
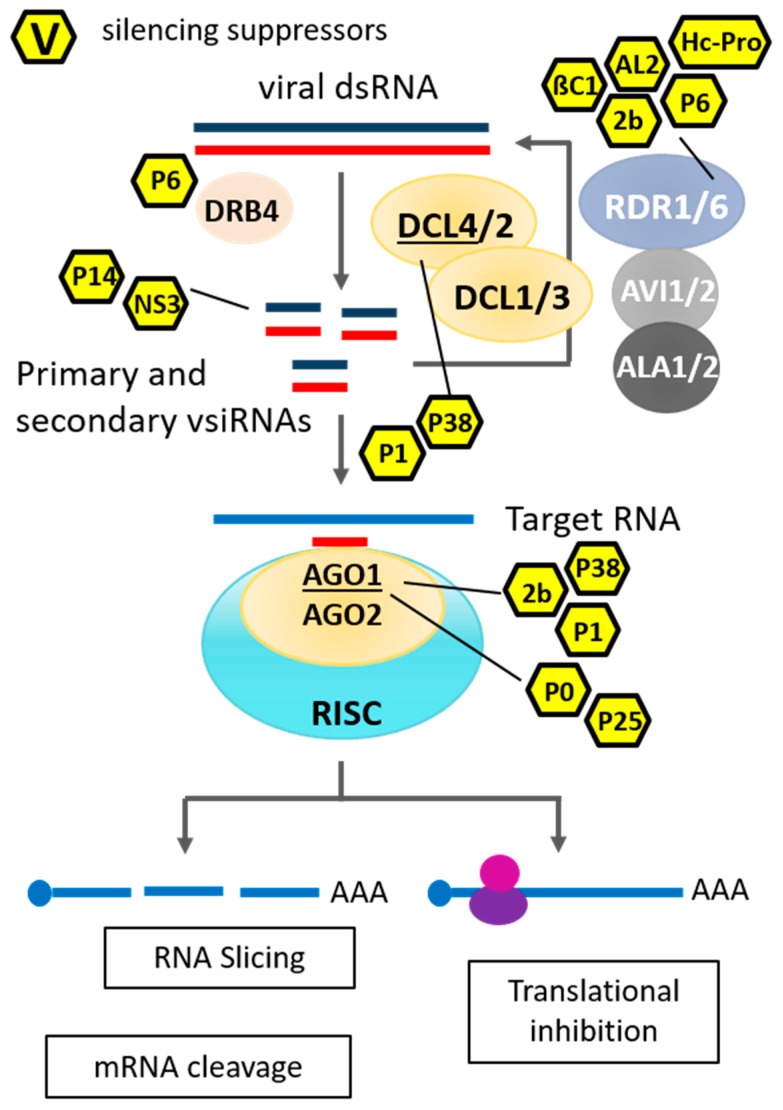
Antiviral RNA silencing starts with the initial processing or cleavage of a precursor viral dsRNA into short 21–24 nucleotide vsiRNA duplexes by RNaseIII-like enzymes called DCLs. Double-stranded siRNAs are incorporated into an RNA-induced silencing complex RISC, and the activated RISC subsequently unwinds the vsiRNA, thereby generating an antisense (or guide) strand that targets complementary mRNA transcripts via base-pairing interactions. Subsequent degradation of the targeted viral mRNA or inhibition of translation can interfere with protein biosynthesis.

RNA silencing is the best-studied antiviral defence mechanism in plants [46,47]. Notably, a few studies reported a similar antiviral function in mammals [48,49,50,51]; however, its diversity depends mainly on the existence of multiple copies of the core RNA-silencing pathway components, which, presumably, are the result of gene duplication followed by specialization [52,53]. For example, the model plant *Arabidopsis thaliana* has four DCLs, ten AGOs and six RDRs, which are involved in different silencing-related pathways [46]. In Arabidopsis, DCL3, DCL4 and DCL2 are important for virus-induced RNA silencing, and DCL3 is also crucial against DNA viruses [54,55,56,57]. AGO1, AGO2, AGO4, AGO5, AGO7 and AGO10 of Arabidopsis are the central players in antiviral RNA silencing [58]. RDR1, RDR2 and RDR6 are shown to display antiviral activity amplifying virus-derived small interfering RNAs or function as a silencing signal [59] (Figure 1). Besides RDR mediation, amplification of small-interfering viral RNA (vsiRNA), short distance (cell-to-cell) as well as long distance (e.g., phloem) spreading of silencing signals is an important aspect of PTGS for establishing systemic antiviral immunity. The reception of long-distance mRNA PTGS and the generation of secondary siRNAs in the recipient cell/tissue requires coordinated activity of a set of proteins. These proteins include AGO1/AGO2 [60], DCL2 [61], RDR6 [52] and SGS3 (SUPPRESSOR OF GENE SILENCING 3) [62], which orchestrate to facilitate the silencing signal amplification process. Notably, proteins associated with TGS pathways also contribute to systemic PTGS, as RNA polymerase IVa, RDR2, AGO4 and DCL3 are involved in the reception of long-distance silencing [63,64].

Interestingly, there is increasing evidence that PTGS and TGS mediated by endogenous sRNAs are key regulators of PTI and ETI [65,66].

Given the complex regulatory mechanisms of antiviral plant defence responses, viruses often cause severe diseases with immense economic losses in agricultural production, suggesting that they have evolved an efficient counter-defence to circumvent plant antiviral immunity [67]. Consistent with this notion, viruses possess a huge repertoire of proteins, which act as RNA-silencing suppressors to dampen host antiviral defences. Viral suppressors of RNA silencing (VSRs) are phylogenetically unrelated, mostly multifunctional proteins that antagonize multiple steps of the RNA-silencing pathway, including (i) impairment of viral siRNA biogenesis by inhibiting DCL proteins and/or the activity of cofactors, (ii) sequestration of dsRNA/siRNA, (iii) promotion of AGO protein destabilization prior to RISC formation or (iv) transportation of the mobile silencing signal into the peroxisomes to disable plant defence [57,68]. Additionally, some VSRs play a pivotal role in replication, assembly or movement of viruses [12]. VSRs have been identified from almost all plant virus genera [57,67]; nevertheless, the multi-functionality of these proteins and the biochemical processes in which they may be involved, such as fine-tuning the plant-virus interaction, hampers the unravelling of their exact mode of action.

To overcome RNA-silencing suppression caused by VSRs, plants have evolved specific defence mechanisms [32], supporting the hypothesis of a molecular arms race between VSRs and RNA-silencing pathway genes [69]; however, studying the diversity of VSRs and the co-evolution between plants and viruses will increase our understanding of plant molecular biology as well as biochemical and cellular activities [70]. Importantly, a lack or inactivation of VSRs leads to the recovery of plants from viral infections [40,71]. For detailed information on the role of VSRs in host-virus interactions, the readers are referred to recent reviews [57,72,73].

## 3. Improvement of Plant Immunity Using RNA Silencing-Based Plant Protection Strategies

Over the last decade RNA silencing has emerged as a powerful genetic tool for scientific research. In addition to fundamental research for the assessment of gene function, RNA-silencing technology has been employed to develop plants with improved resistance to biotic stresses based on their function to provide protection from invasion by foreign nucleic acids, such as viruses (reviewed by [74,75]). This natural phenomenon can be used to control agronomically relevant plant diseases, based on the demonstration that in vitro feeding of dsRNA can signal PTGS of target genes in various plant pests and pathogens [76,77,78,79]. Indeed, expression of such dsRNAs in the corresponding host plant conferred protection from predation or infection [80]. This biotechnological method, termed host-induced gene silencing (HIGS), has emerged as a promising alternative in plant protection because it combines high selectivity for the target organism with minimal side effects, as compared with chemical treatments. In previous studies, we have demonstrated that transgenic Arabidopsis and barley (*Hordeum vulgare*) plants, expressing a 791 nucleotide (nt) dsRNA (CYP3RNA) targeting all three *CYP51* genes (*FgCYP51A*, *FgCYP51B*, *FgCYP51C*) in *Fusarium graminearum* (*Fg*), inhibited fungal infection via HIGS [81,82].

In the following section, we will review recent applications of the HIGS strategy to engineer virus resistance in crop plants. We will not discuss HIGS strategies against non-viral pathogens, as this topic was reviewed previously [80,83].

## 4. RNA Silencing-Based Crop Protection Against Viruses

Many viruses are transmitted via vectors or physical wounds, multiply rapidly and spread across the same or different plant species. Plants naturally resist against viruses using an RNA-silencing mediated defence, which is often not sufficiently effective to stop viral infection completely because siRNA molecules complementary to viral sequences usually appear at later stages of infection [84,85]. Mimicking the RNA-silencing mechanism in planta to generate siRNAs using genetic engineering and biotechnology approaches may help to induce resistance against viruses even before the onset of infection. Since RNA silencing has been shown to synergize with plant innate immunity pathways, integration of different defence layers is expected to ensure a robust defence response against plant viruses [32].

The inoculation of plants with attenuated strains of viruses or viroids that confer cross-protection against more virulent strains to reduce yield losses in cash crops, such as potato or tomato, was already recognized more than half a century ago [86]; however, the first report indicating that plants can be genetically transformed for resistance to virus disease development was published in 1986 [87]. The authors generated a chimeric gene encoding the tobacco mosaic virus (TMV) (species *Tobacco mosaic virus*) CP that was introduced into tobacco cells through *Agrobacterium tumefaciens*-mediated transformation [87]. Transgenic plants showed suppression of symptom development after infection with TMV. At that time, the underlying mechanism of transgene-induced viral resistance and cross-protection remained elusive. More than three decades later, the induction of RNA silencing by tentransgenic expression of virus-derived dsRNA in planta has been successfully implemented to control plant viral diseases.

Since the first publications in the early 1990s [88], a vast number of studies on host-derived gene silencing in plant-virus interactions have been conducted (Table 1). In 2011, there were more than 30 new reports on RNA-silencing technology for controlling a wide array of viral plant diseases. Early in 2012, studies revealed the efficiency of RNA silencing to control viral diseases, e.g., in: *Solanum tuberosum* [89], *Cucumis melo* [90], grapevine [91], banana [92] and rice [93]. In this review, we present an overview on RNA silencing mediated control of plant viral disease in transgenic crop plants. In the following sections, we will highlight a few studies representative for the significant progress that was achieved in this area, and we will discuss transgenic virus resistance of various crop plants for major plant families, such as Solanaceae, Cucurbitaceae, Fabaceae, Poaceae, Euphorbiaceae and tropical fruits. Due to the vast number of publications reflecting the rapid progress of RNA silencing and its great relevance for agriculture, it is not possible to cover all studies in the following sections. Nevertheless, a complete overview is given in Table 1.

## 5. RNA Silencing-Mediated Viral Resistance in Solanaceae—*Tobacco, Tomato and Potato*

The economic importance of plant viruses infecting solanaceous hosts like tobacco is reflected by the vast number of HIGS studies related to the species *Nicotiana benthaminana* and *Nicotiana tabacum* (Table 1). Rigid, rod-shaped (+) ssRNA viruses of the genera *Tobamoviruses* and *Cucumovirus* cause serious agronomical losses by damaging the leaves, flowers and fruits of their hosts (including tobacco, tomato, cucumber and pepper). Diseases caused by the TMV and cucumber mosaic virus (CMV, species *Cucumber mosaic virus*) are found worldwide; thus, scientists used genetic engineering to introduce TMV and CMV resistance. The utility of virus-derived transgenes expressing dsRNA that mediate viral RNA silencing was demonstrated by different groups (Table 1). They showed that tobacco plants transformed with inverted repeated sequences of the partial TMV movement protein (MP) gene and the partial sequence of CMV replication protein (RP) gene exhibited PTGS of the corresponding genes [94]. The transgenic plants exhibited complete resistance to TMV or CMV infection. Moreover, the authors proved that the silencing was stably inherited through self-pollination in T4 progeny and that viral resistance was unaffected by low temperature (normally compromises siRNA–mediated gene silencing [166]) [94].

A key step in developing a successful RNA-silencing strategy is the identification of suitable target genes in the infectious agent. Whereas approximately 20% of the publications demonstrated that the replicase gene represents a prominent target, every third study chose partial- to full-length sequences of the CP gene to confer viral disease resistance (Table 1). Transgenic tobacco plants that express dsRNA homologous to the CP gene of TMV and CMV were proven to trigger RNA silencing of the corresponding viral genes [95,96,167]. Moreover, the authors found that the number of siRNAs correlates with the degree of resistance [95]. Interestingly, they showed that only 17% of the transgene-expressing plants generated substantial amounts of siRNAs that confer CMV resistance. Several years later, the same authors addressed the question whether multiple transgene copies coincided with the processing of hpRNA to siRNA and the occurrence of resistant phenotypes [167]. They found no significant correlation between the resistance and the copy number of the transgene and consistent with another study [94]; therefore, structural characteristics of the RNA-silencing construct [148,149], the locus of transgene integration [168,169] and the promotor used for transgene expression [170] as well as undesired transgene silencing provoked by using transgenic technologies [171] may account for RNA-silencing effectiveness.

However, target gene silencing is correlated with the number of siRNAs generated from a dsRNA precursor; thus, it can be used as a molecular marker to predict success in attempts to engineer virus resistance by dsRNA. Previously, it was shown that dsRNA derived from hpRNA constructs of different lengths led to divergent resistance phenotypes [98], which is consistent with our own work related to fungal gene silencing. Using barley as a cereal model, we found that dsRNA constructs targeting two *FgCYP51* genes inhibited fungal growth more efficient than single constructs, although both types of dsRNAs decreased fungal infections [unpublished]. Based on these findings and combined with our recent study on the efficacy of dsRNAs with increasing length in RNA silencing of the Fusarium *CYP51* genes, we anticipate that constructs of >400 bp in length were more efficient because the number of siRNAs derived from those longer constructs are higher [unpublished].

Whereas most studies used virus-derived dsRNAs or dsRNAs derived from hpRNAs processed by host RNA silencing machinery into siRNA to develop virus-resistant plants, miRNA-based approaches have been explored for engineering plant virus resistance [91,97,116,161]. The first study that investigated the possibility of modifying plant miRNA sequences to target specific viral transcripts was published in 2006 by Niu et al. [161]. They used a 273-bp backbone of the miRNA precursor pre-miRNA^159a^ to generate artificial pre-miRNA^159a^ containing sequences complementary to the plant viruses turnip yellow mosaic virus (TYMV, species *Turnip yellow mosaic virus*) and turnip mosaic virus (TuMV, species *Turnip mosaic virus*) [161]. They designed their amiRNAs to target the sequence of two silencing suppressors, P69 of TYMV and HC-Pro of TuMV. Transgenic Arabidopsis plants that were expressing amiR-P69^159a^ and amiR-HC-Pro^159a^ exhibited resistance to both viruses. This pioneering work was subsequently confirmed by two studies using amiRNAs to target the VSR 2b and 2a of CMV in tobacco [97] and tomato [116].

The authors claimed that the use of amiRNAs has several advantages: (i) high specificity, thus diminishing off-target effects, (ii) ease of amiRNA design and (iii) applicability to diverse viral pathogens. Consistent with this, the design of synthetic RNAs and amiRNAs has been reported as an effective tool for functional gene studies in plants as well as for therapeutic approaches in animal and human cells [172,173].

The use of target-specific dsRNA or amiRNA as an anti-viral agent offers unprecedented potential as a new plant protection strategy; therefore, successful field application will require optimization of an RNA-silencing construct design necessary to maximize the efficacy of the RNA-silencing-based pathogen control. Consistent with this, the RNA-silencing construct design can be directed either to combine high selectivity for the target organism with minimal side effects on beneficial microorganisms, or generating chimeric sequences to achieve broad-spectrum resistance to multiple viral pathogens.

Consistent with the idea of parallel or simultaneous silencing of different targets by using a single chimeric transgene construct, a recent study demonstrated that fused viral coat protein coding sequences from potato virus X (PVX), potato virus Y (PVY) and potato virus S (PVS) conferred simultaneous resistance against all three RNA viruses in potato [122]. The authors fused 180 bp (PVX), 240 bp (PVY) and 180 bp (PVS) fragments for a total of 600 bp sequence for developing broad-spectrum virus resistance in transgenic potato plants. Simultaneous resistance to multiple viruses was previously reported by the transgenic expression of fused-tandem, repeat, virus-derived dsRNAs [89,102,174]. However, resistance to mixed infection by two different potato viruses can be achieved without using fused-tandem constructs [175].

Although dsRNA as well as hpRNA which represent the intermediate forms during viral replication, are the key triggers of RNA-silencing machinery [176,177] and are, thus, expected to exhibit stronger virus resistance [178], earlier studies were performed using antisense RNA constructs [88,110]. For example, transgenic tobacco plants expressing an antisense RNA that compromises the complete coding sequence of the *AL1* gene encoded in members of the species *Tomato golden mosaic virus* in the genus *Geminivirus* reduced symptom development and viral replication [88]. The authors argued that the *AL1* gene is required for DNA replication and, thus, together with its conservation in all *Geminiviridae*, makes it a promising target. Moreover, the transgenic cassette compromised the 5′ sequences of two other open-reading frames, *AL2* and *AL3*, resulting in a 1258-bp fragment. The authors found that the lines expressing the most antisense RNAs exhibited the highest resistance. Importantly, the copy number of the integrated DNA did not correlate with the level of resistance or antisense RNA [88]; however, the percentage of plants that showed a reduction in symptom development after inoculation with TGMV differed between lines transformed with the same construct. Thus, this, together with the results from other studies (reviewed above), points to the variation in gene expression that can be explained, for example, by the transcriptional activity of the region surrounding the integrated transgene.

## 6. RNA-Silencing-Mediated Viral Resistance in Cucurbitaceae—*Cucumber*, *Melon* and *Watermelon*

Cucurbit crops, such as melon, cucumber, squash/pumpkin and watermelon, are susceptible to at least 35 viruses [179] that can cause massive damage up to total loss [180]. Improvement of viral resistance relies mainly on biotechnological approaches, such as genetic engineering.

Previously, we have demonstrated that transgenic Arabidopsis and barley (*Hordeum vulgare*) plants, expressing a 791 nt dsRNA (CYP3RNA) that targets all three *CYP51* genes (*FgCYP51A*, *FgCYP51B*, *FgCYP51C*) in *Fusarium graminearum* (*Fg*), inhibited fungal infection via HIGS [82]. Concurrently, the HIGS technology enables us to generate constructs that are highly specific for the targeted genes, preventing side effects on other (beneficial) microbes and host plants; however, the homology of CYP3RNA to the *CYP51* genes raise the possibility that this HIGS strategy can be used to control a wide range of fungal pathogens. Consistent with this idea, the silencing of a single-target gene can confer broad-spectrum and durable resistance to multiple viral pathogens of the cucurbitacous host melon (*Cucumis melo* L.) [90].

Virus resistance can be achieved through the absence of host factors, known as susceptibility factors, that are required for the virus to complete its biological cycle [181]. Moreover, it was shown that different viruses have common susceptibility factors, thus indicating that RNA-mediated silencing of those host factors could confer broad-spectrum disease resistance. In melon, the eukaryotic translation initiation factor Cm-eIF4E represents such a common susceptibility factor [181]. In 2012, Rodríguez-Hernández et al. [90] generated transgenic melon plants expressing a hpRNA that induced silencing of *Cm-eIF4E*. The authors hypothesized that Cm-eIF4E may control the susceptibility of a broad range of viruses; therefore, they challenged these transgenic melon plants with eight agronomically important melon-infecting viruses and identified that they were resistant to cucumber vein yellowing virus (CVYV), melon necrotic spot virus (MNSV), Moroccan watermelon mosaic virus (MWMV) and zucchini yellow mosaic virus (ZYMV) [90]. A previous report has shown that silencing of the VSR HC-Pro from ZYMV conferred resistance not only to ZYMV but also to the papaya ringspot virus and watermelon mosaic virus (WMV) in cucumber and melon [126]. This finding is based on co-silencing effects related to sequence homologies between the *HC-Pro* genes of those three viruses. Recently we found that fungal resistance induced by CYP51-dsRNAs (targeting the fungal ergosterol biosynthesis) in Arabidopsis mirrors the co-silencing of non-target *FgCYP51* genes [unpublished]. Overall, these results suggest that resistance conferred by single dsRNA constructs can be mediated by co-silencing effects on non-targeted genes of a wide range of pathogens.

However, multiple-virus resistance in transgenic plants can also be achieved by using chimeric transgene constructs composed of viral gene segments that induce PTGS without the expression of dsRNA trigger molecules [127,182,183,184,185,186]. The underlying mechanism is based on transgene silencing in a length-dependent manner that is independent of the ability to provide homology-dependent trans-inactivation of a homologous, incoming virus [187]. The authors found that the expression of large segments (387–453 bp) of the nucleocapsid (*N*) encoding gene of the tospovirus were silenced in transgenic tobacco plants mediating tomato spotted wilt virus (TSWV) resistance through PTGS. Importantly, small *N* gene segments (92–235 bp) were ineffective in conferring resistance unless they were transcriptionally fused to the non-target *GFP* gene DNA. The authors argued that the inability of a small *N* transgene alone to induce homology-dependent virus resistance was because they are incapable of inducing gene silencing [187]. Importantly, the same authors provided the hint that any viral sequence with a minimum length of 59–110 bp could confer RNA silencing-mediated resistance when fused to a transcribed DNA, designated as a silencer DNA (in this case a green fluorescent protein (GFP) gene) [182]. However, in a subsequent study they replaced the silencer GFP DNA with the full-length *CP* gene of turnip mosaic virus (TuMV) linked to 218 or 110 bp *N* gene segments for the transformation into *Nicotiana benthamiana* [188]. Transgenic lines with the 218 bp *N* gene segment linked to the TuMV *CP* gene exhibited TSWV resistance. More than one decade later, they transferred their strategy for engineering multiple-virus resistance in transgenic watermelon [127] and generated a chimeric transgene consisting of three viral *CP* gene segments of CMV, cucumber green mottle mosaic virus (CGMMV) and WMV fused together and cloned into a plant transformation vector carrying a silencer DNA comprising the middle half *N* gene of watermelon silver mottle virus (WSMoV), resulting in a transgene length of 1.7 kb. The authors identified two transgenic lines (*R*_0_ Lines 6 and 14) that showed resistance to all three viruses individually and mixed infection [127]. Together, these studies showed that PTGS is the underlying mechanism for the multiple-virus resistance that can be triggered without the expression and formation of transgene-derived dsRNAs.

## 7. RNA-Silencing-Mediated Viral Resistance in Fabaceae—*Soybean*

Conventional soybean protection measures based on managing virus vectors were less efficient [189]; therefore, RNA-silencing technology represents a promising alternative. A recent study demonstrated that RNA-mediated silencing of the soybean mosaic virus (SMV) *P3* cistron induced resistance to five different SMV strains, the soybean-infecting bean common mosaic virus (BCMV) and the WMV in transgenic soybean [133]. *Soybean mosaic virus* belongs to the virus family *Potyviridae* of which the genus *Potyvirus* contains the most numerous and most important plant viruses. SMV is the most devastating viral pathogen in soybean and can cause severe yield losses [190]. The P3 protein is involved in virus replication, movement, pathogenesis and SMV virulence [191,192,193] and, thus, represents a suitable target for RNA-silencing-mediated approaches [133]. The P3 cistron-silencing mediated by a 302-bp inverted repeat (IR) provoked significantly enhanced SMV resistance under field conditions over three years. However, SMV resistance can also be achieved by expression of viral genes and sequences in transgenic soybeans [194,195,196,197].

Further studies reported successful engineering of viral resistance in soybean using RNA-silencing strategies [128,129,130,131,132,198]; thus, SMV resistance was achieved by silencing the VSR HC-Pro [130,132] and the *CP* gene [199]. Multiple-virus resistance was conferred by expressing a transgene designed to express several shorter IRs [129]. The authors chose specific, highly conserved sequences of less than 150 bp derived from three viruses, alfalfa mosaic virus (AMV), bean pod mottle virus (BPMV) and SMV to assemble the short IRs. Transgenic lines exhibited simultaneous resistance to these viruses, blocking systemic spread and eliminating seed mottling. The authors argued that their strategy of construct assembly makes it easy to incorporate additional IR sequences of viral origin, thus, conferring resistance to a wide range of soybean-infecting viruses [129]. The same authors conducted another study to compare virus-resistant transgenic soybeans with their non-transgenic counterparts to verify whether biologically important changes occurred due to IR insertion [200]. The authors followed the concept of “substantial equivalence” developed by the Organisation for Economic Co-operation and Development (OECD) in 1993 [201] and further elaborated by FAO/WHO [199,200]. Their substantial equivalent analysis comprised: (i) proximate analyses, including moisture, ash content, crude protein, crude fat and carbohydrates; (ii) the content of 18 amino acids; (iii) fatty acid composition; (iv) the analyses of two important antinutritional factors (lectin and trypsin inhibitors); (v) the contents of isoflavones, including daidzin, glycitin, genistin, daidzein, glycitein and genistein, of transgenic soybeans and non-transgenic counterpart seeds. Their results showed that the RNA-silencing-mediated virus-resistant transgenic soybeans are as safe and nutritious as their traditional counterparts [200]. The authors claimed their findings can serve as baseline information for future generations of RNA-silencing-mediated virus-resistant transgenic crops.

To use the sense *CP* gene to direct RNA silencing of viral target genes can induce PTGS, although the complementarity to RNA transcripts is not given. Moreover, most studies were using sense gene sequences as transgenic controls (Table 1). Interestingly, transgenic soybeans expressing the sense *CP* gene of the soybean dwarf virus (SbDV) exhibited SbDV-CP-specific siRNAs, and those plants remained symptomless after SbDV inoculation [198]; however, the underlying mechanisms remain elusive.

## 8. RNA-Silencing-Mediated Viral Resistance in Poaceae—*Rice*

RNA-silencing-mediated biotechnological approaches to generate genetically engineered cereals, such as maize, rice, wheat and barley, represent a promising tool to improve plant immunity towards viral infections. RNA-silencing-based plant protection strategies provide a promising alternative to genetic resistance approaches, especially if there are no naturally occurring genes that confer virus resistance to e.g., rice dwarf virus (RDV), a dsRNA virus of the family *Reoviridae* which causes severe disease in rice crops. Symptoms of RDV include stunting of plant growth and white chlorotic spots on leaves, leading to decreased grain yields of their hosts. RDV is transmitted exclusively by leafhoppers (*Nephotettix* spp.). The genome of RDV consist of 12 segments of dsRNA, designated S1-S12 that encode seven structural and five non-structural proteins [202]. Shimizu and co-authors showed in 2009 [138] that RNA silencing of the non-structural RDV protein Pns12 confers resistance to RDV by insertion of a IR construct that led to the formation of a 500-bp Pns12-complementary dsRNA in transgenic rice plants [138]. Moreover, they generated transgenic rice plants carrying an IR construct specific to the Pns4 non-structural protein to compare resistance phenotypes of both RNA-silencing targets. Whereas Pns12-dsRNA expressing plants exhibited complete RDV resistance, Pns4-dsRNA plants displayed incomplete resistance. The authors argued that targeting Pns12, which plays a crucial role in viral replication at an early stage of infection [203], was more effective for controlling RDV than targeting the phosphoprotein Pns4, which is localized around the viroplasm matrix, forming minitubules, and is expressed at a relatively late stage of viral infection [204]. Importantly, the same authors showed that targeting of viroplasm matrix proteins represents an applicable strategy to control viruses of the family *Reoviridae* [93,141]. In the case of the genus *Fijivirus*, RNA silencing of viroplasm protein P9-1 of rice black streaked dwarf virus (RBSDV) induced virus resistance in rice [141]. In another study, they showed that IR-derived dsRNA that corresponds to the non-structural viroplasm protein Pns9 of rice gall dwarf virus (RGDV) confers strong resistance to RGDV infection in transgenic rice plants [93]. Consistent with their study published in 2009, Shimizu and co-authors further demonstrated that the identification of suitable potent viral target genes is essential to guarantee strong resistance effects mediated by RNA silencing-based plant protection approaches, as not all RNA-silencing constructs exhibit equal effectiveness in preventing virus infection [140]. They attempted to develop rice stripe virus (RSV) resistance by targeting seven different RSV proteins (pC1-4 and p2-4). The targets *pC3* and *pC4*, which encode a nucleocapsid protein and a movement protein of RSV, respectively, were more resistant to RSV infection compared with RSV-resistant rice cultivars cv. Musashikogane and Sainokagayaki. Similar results were obtained using RNA silencing of NP *pC5* gene and MP *pC6* gene of rice grassy stunt virus (RGSV) that displayed strong RGSV resistance indicating that NP and MP represent promising targets to control viral disease caused by members of the genus *Tenuivirus* [142]. Targeting the *pC1* gene, which encodes a putative RNA-dependent RNA polymerase, also conferred high level resistance to RSV compared with susceptible wild-type rice [143]. Transgenic rice plants that harbour p2-(protein of unknown function) and p3 (VSR)-specific RNA-silencing constructs exhibited moderate resistance to RSV infection. By contrast, the IR constructs that correspond to the *pC2* gene (encodes a glycoprotein of unknown function) as well as the *p4* gene (encodes a non-structural protein of unknown function) failed to induce enhanced disease resistance, underlining that not all RNA-silencing constructs establish equal resistance phenotypes [140].

## 9. RNA-Silencing-Mediated Viral Resistance in Poaceae—*Wheat and Barley*

Wheat streak mosaic virus (WSMV) (genus *Tritimoviruses*; family *Potyviridae*) causes yellowish-orange streaks on leaves of their hosts, which are restricted to the *Gramineae* family, and is transmitted by eriophyid mites. WSMV is one of the most important destructive viral diseases of wheat causing severe yield losses [205,206]. There are two reports demonstrating that RNA-silencing-mediated virus control represents a promising alternative to natural resistance strategies [143,144]. The authors selected the nuclear inclusion protein *NIa* gene of WSMV as an RNA-silencing target. Notably, transgenic wheat plants harbouring a single insertion of the *Nia* transgene were immune to WSMV infection [143,144].

Another study in barley used the RNA-silencing strategy to control barley yellow dwarf virus (BYDV) [146], which occurs throughout the world and affects a wide range of gramineous hosts. Yield losses caused by the impairment of tillering, flowering and kernel production range from 5%–30% in wheat and barley in years of BYDV outbreaks [207]. Barley plants can be protected from BYDV infection by transformation with hpRNA constructs expressing dsRNA complementary to the polymerase gene of BYDV [146]. Notably, BYDV-PAV immunity was not affected by cereal yellow dwarf virus-RPV co-infection based on the finding that some viruses have the capacity to enhance replication and spread of the co-infecting viruses and to inactivate PTGS [208]. The authors presumed that using hpRNA technology to generate transgenic BYDV-immune barley plants might have great potential for field trials [146].

## 10. RNA-Silencing-Mediated VIRAL Resistance in Euphorbiacea—*Cassava*

Cassava production in sub-Saharan Africa is limited by two devastating viral diseases: Cassava mosaic disease (CMD) and cassava brown streak disease (CBSD). CMD is widespread over the East African countries, where 150 million people depend on the tropical staple cassava as their largest source for carbohydrates [209]. CMD is caused by *African cassava mosaic virus* (ACMV), which can result in severe losses of approximately 20%–30% of the annual production [209]. RNA-silencing technology has been extensively used to bring effective solutions to viral diseases that diminish cassava yields and reduce farmer incomes in East Africa. Towards this, a collaborative program named the Virus Resistant Cassava for Africa (VIRCA) project was founded in 2005 [209]. The VIRCA strategies rely on RNA-silencing-mediated viral disease control through transgenic expression of RNA-silencing triggers. Consistent with this, there are several studies demonstrating CMD and CBSD resistance using RNA-silencing technology (Table 1).

For example, CBSD resistance was achieved by targeting the full-length CP of cassava brown streak Uganda virus (CBSUV) through transgenic expression of RNA silencing constructs in cassava [153]. Previously, the authors tested three RNA-silencing constructs consisting of full-length (894 bp), N-terminal (397 bp) and C-terminal (491 bp) portions of the CP gene of CBSUV in transgenic *Nicotiana benthamiana* plants and identified FL-CP as the most efficient construct [105]. However, the same authors underlined the significance of their findings by testing seven FL-CP-siRNA-producing lines under conditions of naturally vectored disease pressure under confined field trials [154]. They demonstrated that field-grown transgenic cassava plants remained symptom-free over the trial period of 11 months, underlining the great potential RNA-silencing technology holds for reaching goals of the VIRCA project. Furthermore, they confirmed RNA-silencing-mediated CBSD resistance through a typical vegetative propagation cycle [155]. Their studies provided significant progress towards the development and delivery of CBSD-resistant planting materials for farmers’ use in East Africa. Additionally, Vanderschuren et al. [152] strengthened the potential of RNA-silencing technology to control CBSV in the West African cultivar TME7. Recently, Beyene et al. (2017) [156] genetically modified the Ugandan farmer-preferred cassava cultivar, TME 204, with an improved RNA-silencing construct, consisting of the CP sequences of Ugandan cassava brown streak virus (UCBSV) and CBSV fused in tandem. The resulting transgenic lines exhibited high levels of resistance to both viruses [156] under subsequent field trials in Uganda and Kenya [151].

ACMV, as one of the causal agents of CMD, was also a focus of the VIRCA project [209]. Pioneering work towards RNA-silencing-mediated generation of CMD-resistant cassava was performed in collaboration between the labs of Wilhelm Gruissem (Switzerland) and Peng Zhang (China) [147,148,149,152]. First, they used antisense RNAs against viral mRNAs, encoding essential non-structural proteins required for viral replication and transcriptional regulation of ACMV, such as Rep (AC1), TrAP (AC2) and Ren (AC3) [147]. Towards this, they inserted full-length sequences of those target genes in antisense orientation, engineering ACMV-resistant transgenic cassava, with antisense-AC3 lines exhibiting the highest level of resistance. However, the use of sense and antisense RNA strategies produced lines showing large variations in resistance levels under high viral infection pressure [147,210]. The authors argued that using hp-derived dsRNA approaches are more efficient based on siRNA quantities. Consistent with this idea, they observed a dose-dependent sRNA-mediated ACMV resistance in transgenic cassava expressing a short (155 bp) hp construct [149]. Whereas other RNA-silencing-based strategies rely on the use of long introns and hp arms longer than 400 bp to produce intron hp-derived sRNA (Table 1), they provided a proof-of-concept that short introns and short hp arms combined with a strong promotor (p35s) might represent the most efficient strategy to reach the highest levels of target-specific sRNAs [149]. Notably, they found a substantial quantity of sRNA derived from the base-paired region of the hp that were longer compared to the known 21–24 nt RNA-silencing-related sRNAs. Whether these long sRNA contribute to target gene silencing and virus resistance remain unknown.

Interestingly, in 2007, they expanded their former RNA-silencing strategies in controlling DNA viruses of the family *Geminiviridae* through expression of dsRNA homologous to viral non-coding sequences [148]. They designed an RNA-silencing construct that consisted of the bidirectional promotor and the common region sequence of ACMV. Although they identified ACMV-resistant lines containing siRNAs derived from non-coding sequences, they concluded that transformation efficiencies of transgenic cassava plants harbouring siRNAs originating from ACMV-dsRNA targeting viral coding sequences were much higher [148]. Whether the low plant regeneration frequency was due to the transgene they transformed remained speculative; however, their results principally suggest that RNA-silencing technology can be expanded to target the entire genome of a DNA virus to confer disease resistance.

## 11. RNA-Silencing-Mediated Viral Resistance in Fruits—*Citrus*

The viral disease of citrus represents a serious threat to farmers in all citrus-growing areas of the world. Citrus tristeza virus (CTV) can cause severe losses of fruit quantity and quality of its hosts, primarily orange, grapefruit and lime [211], along with the death of the tree. The group of Leandro Peña in Spain provided huge progress in engineering CTV-resistant Mexican lime [158,159,212]. Their RNA-silencing strategies rely on the silencing of CTV-VSR based on their former work where they identified p23 as an important CTV pathogenicity determinant through its ectopic expression that induced viral-like symptoms [213,214]. Surprisingly, five of the p23-transgenic lines displayed characteristics of PTGS and TGS, such as the accumulation of p23-specific siRNAs, low levels of the corresponding mRNA as well as transgene silencing resulting in a CTV-immune phenotype [212]. Based on this finding that Mexican lime plants transformed with a silenced p23 transgene showed resistance to CTV infection, they overexpressed the 3′-terminal 549 nt region of the *p23* gene together with the 3′UTR in sense, antisense and hp formats to generate RNA-silencing-mediated CTV resistance [158]. Unexpectedly, they found that transgene-derived siRNA levels varied in different transformants irrespective of their response to CTV, indicating that high abundancies of transgene-derived siRNA are not necessarily correlated with CTV resistance [158]. The authors suggest that this lack of correlation is presumably specific for RNA viruses, further arguing that there is a siRNA threshold below which the virus can overcome resistance, which is consistent with previous reports [215,216]. However, in a subsequent study, they extended their RNA-silencing approach and transformed Mexican lime with an intron hp vector carrying full-length sequences of two more CTV-VSR, p20 and p25, providing proof-of-concept that simultaneous targeting of three VSRs confer transgenic resistance to CTV [159].

*Citrus psorosis virus* (CPsV), an (−) ssRNA virus, is another causal agent of a serious disease affecting citrus. The group of María Laura García in Argentina developed CPsV-resistant transgenic orange plants via RNA silencing of the *CP* gene [160]. Notably, they obtained similar results as the group of Leandro Peña working on CTV-resistance in which the level of accumulated siRNAs did not correlate with the degree of virus resistance among different lines/transformants. Those results further support the notion that virus resistance cannot be predicted only by the presence of high amounts of transgene-derived siRNAs, whether this is a general aspect of using RNA-silencing technology or something specific to RNA silencing pathways in citrus trees remains to be elucidated.

## 12. Outlook: Non-GMO RNA Spray Confer Virus Resistance

While all the reviewed studies provided proof-of-concept that RNA-silencing-based plant protection is an effective strategy for controlling diseases caused by viral pathogens, the broad applicability of HIGS remains questionable due to fact that generation of genetically modified (GM) crops is time-consuming and still poorly accepted in many European countries. Moreover, it relies on the transformability of a plant, thus, limiting or making it an exclusive approach for transformable crop plants only. Whereas a great number of studies have been published on HIGS-mediated silencing of target genes in pathogenic viruses (Table 1), silencing of such targets through exogenously applied dsRNA has been described in only a few studies [217,218,219,220,221]. The induction of virus resistance by exogenous application of dsRNA has been reviewed recently [for details, see 188]. Therefore, a breakthrough was achieved by showing that inhibitory dsRNA is effective upon spray application. Recently, we have shown that spray applications of CYP3RNA also protect barley from fungal infection via a process termed ‘spray-induced gene silencing’ (SIGS) [222]. In the same year, Konakalla et al. [223] showed that exogenous application of dsRNA targeting the VSR *p126* gene and *CP* gene of TMV conferred virus resistance in tobacco [223]. Importantly, authors confirmed the spreading of dsRNA from local to systemic tissue by one hour after rub-inoculation of dsRNA using semi-quantitative RT-PCR. Moreover, they showed that p126 dsRNA levels continuously decreased in the local (treated) tissue from 3 dpi to 9 dpi where dsRNA was no longer detectable. This is consistent with our own work, which showed movement of sprayed dsRNA from barley leaves over stems to the root tissue within three days after spray treatment (unpublished data). In 2018, Kaldis et al. [224] showed that exogenously applied dsRNA derived from the *HC-Pro* and *CP* genes of ZYMV protect watermelon and cucumber against ZYMV and spread systemically over long distances in cucurbits.

Given the significance of these recent findings, subsequent studies were conducted to improve the stability of sprayed dsRNA and to find a system that allows large-scale production of long dsRNA molecules suitable for application in crop protection [225,226]. To provide long-term protection against the targeted viral pathogen the uptake of sprayed dsRNA into plant cells is a critical step together with improving its stability to overcome environmental degradation by UV radiation or simply surface wash-off by rain. Towards this, Mitter et al. [159] used layered double hydroxide (LDH) clay nanosheets as a dsRNA carrier, a technology originally developed for the delivery of siRNA therapeutics to mammalian cells [227,228]. The authors demonstrated that loading dsRNA on LDH prolonged their durability on the leaf surface for 30 days and increased their stability through protection from nuclease degradation [229]. Moreover, they showed uptake of dsRNA into plant cells and induction of endogenous RNA silencing that mediated systemic protection against the targeted VSR *2b* gene of CMV inoculated on cowpea and tobacco [225]. Notably, they proved that the LDH nanocarrier can be completely degraded over time, thus, resulting in slow and sustained release of dsRNA under environmental conditions. However, whereas this study provides a significant step forward in making RNA spray an applicable and sustainable approach towards pathogen and pest control in agriculture, the question is how to produce efficient amounts of dsRNA for spray applications in field trials. Towards this, Manfred Heinlein and his group in France established a dsRNA production system that enables the broad application of dsRNA molecules as a non-GMO crop protection measure [226]. They used the RNA-dependent RNA polymerase of the dsRNA phage phi6 for in vivo synthesis of dsRNA molecules. Towards this, they engineered a phi6-based dsRNA-synthesizing machine within the bacterium *Pseudomonas syringae*. The authors argued exploiting phi6 that naturally converts ssRNA templates into dsRNA would produce sufficient amounts of dsRNA with higher quality and purity compared to dsRNA generation e.g., by enzymatically synthesized ssRNA strands in vitro and their subsequent physical annealing to dsRNA. Indeed, they showed that application of purified in vivo-produced dsRNA to target either the *GFP* gene or the viral *replicase* gene of a GFP-tagged TMV strain provided efficient protection against local and systemic spread of TMV after virus inoculation [226]. Given the significance of their results, this opened the door for large-scale vaccination applications of long dsRNAs to protect crop plants against several pathogens and pests simultaneously. However, cost-effective production methods of dsRNA for commercialisation of exogenous dsRNA application in plant virus control was published previously (for more information, see [229]).

The reviewed studies are of a ground-breaking nature, as they represent significant progress towards establishing an RNA-silencing-based non-GMO crop protection approach using direct-spray applications of dsRNA to target viral pathogens. However, further research is needed to (i) elucidate the mechanistic basis of entry, transport and procession, (ii) solve problems of durability and instability and (iii) implement the large-scale production and purification of exogenously applied dsRNA to make RNA-silencing-based spray approaches for plant protection scientifically and economically achievable.

## Figures and Tables

**Table 1:** Summary of HIGS applied to control viral pathogens. Host plant family, virus species and genome, target genes, type of RNA silencing trigger and construct length are summarized Hairpin RNA (hpRNA); artificial miRNA (amiRNA).

***Solanaceae***	**Virus**	**Abbr.**	**Type**	**Target Gene**	**RNA Type**	**Construct Length**	**Reference**
Tobacco							
	tobacco mosaic virus		(+) ssRNA	Movement protein	IR (hpRNA)	not mentioned	[94]
	cucumber mosaic virus	CMV	(+) ssRNA	Replicase	IR (hpRNA)	not mentioned	[94]
	cucumber mosaic virus	CMV	(+) ssRNA	Coat protein	IR (hpRNA)	718 bp	[95]
	tobacco mosaic virus	TMV	(+) ssRNA	Coat protein	dsRNA	not mentioned	[96]
	cucumber mosaic virus	CMV	(+) ssRNA	VSR 2b	amiRNAs	Precursor miR171a	[97]
	cucumber mosaic virus	CMV	(+) ssRNA	Coat protein	hpRNA		[98]
	plum pox virus	PPV	(+) ssRNA	VSR P1 and HC-Pro	IR (hpRNA)	733 bp (UTR/P1)	[99]
						649 bp (P1/HC-Pro)	
						706 bp (HC-Pro)	
						678 bp (HC-Pro/P3)	
	cucumber green mottle mosaic virus	CGMMV	(+) ssRNA	Coat protein	IR (hpRNA)	not mentioned	[100]
	tomato bushy stunt virus grapevine leaf virus	TBSV	(+) ssRNA	Defective interfering (DI) Movement protein	Virus-derived (DI) RNA	720 bp	[101]
		GFLV	(+) ssRNA				
	potato virus Y	PVY	(+) ssRNA	Coat protein	fused, tandem, IR; 200 bp (from each virus)	600 bp fragment (PVY, PVA, PLRV)	[102]
	potato virus A	PVA	(+) ssRNA	cylindrical inclusion (CI)		1000 bp fragment (PVY, PVA, PLRV, TRV, PMTV)	
	potato leafroll virus	PLRV	(+) ssRNA	Coat protein			
	tobacco rattle virus	TRV	(+) ssRNA	Replicase			
	potato mop-top virus	PMTV	(+) ssRNA	Replicase			
	potato virus Y	PVY	(+) ssRNA	VSR HC-Pro	amiRNAs	Precursor miR159a, miR167b and miR171a	[103]
	potato virus X	PVX	(+) ssRNA	VSR TGBp1, p25 (p25)			
	cassava brown streak Uganda virus	CBSUV CBSV	(+) ssRNA	conserved sequences of P1(CBSV and UCBSV), P3(CBSV and UCBSV), CI(UCBSV), NIb(CBSV and UCBSV),	amiRNA	Precursor miR159a	[104]
	cassava brown streak virus		(+) ssRNA	CP(UCBSV) and the 3′UTR			
	cassava brown streak Uganda virus	CBSUV	(+) ssRNA	Coat protein (CBSUV)	hpRNA (pHELLSGATE)	Full-length; N-term. 397 bp; 491 C-term.	[105]
	cassava brown streak virus	CBSV	(+) ssRNA				
	African cassava mosaic virus	ACMV	ssDNA	DNA-A and DNA-B genome	dsRNA	12 constructs of different length	[106]
	soybean mosaic virus	SMV	(+) ssRNA	Coat protein	hpRNA (pK7GWIWG2)	not mentioned	[107]
	bean yellow mosaic virus	BYMV	(+) ssRNA	Coat protein	hpRNA (pK7GWIWG2)	not mentioned	[107]
	citrus psorosis virus	CPsV	(−) ssRNA	Coat protein and *54K* gene	hpRNA (pHANNIBAL)	372 bp (CP); 436 bp (54K)	[108]
	citrus tristeza virus	CTV	(+) ssRNA	3′ p23 (VSR) and 3′ UTR	hpRNA	900 bp	[109]
	tomato golden mosaic virus	TGMV	ssDNA	Replicase (*AL1*; *AL2*; *AL3* gene)	antisense construct	1258 bp	[88]
	tomato yellow leaf curl virus	TYLCV	ssDNA	Replicase (*C1* gene)	antisense RNA		[110]
	tomato leaf curl New Delhi virus	ToLCNDV	ssDNA	virion-sense gene (*AV2*)	antisense construct		[111]
	cotton leaf curl disease	CLCuD	ssDNA	Replicase (*AC1*)	sense and antisense RNAs	446 bp (AC1(5′half)+AC4(ORF))	[112]
				Transcription activator (*AC2*)		523 bp (AC1 (3′half)+AC2,AC4)	
				Rep enhancer (*AC3*)		510 bp (AC1(97bp) +AC2+AC3)	
	chickpea chlorotic dwarf Pakistan virus	CpCDPKV	ssDNA	Rep gene, large intergenic region (LIR) and part of the MP gene	hpRNA	730 bp	[113]
						5′ Rep, LIR and 5′ MP	
	pepper golden mosaic virus	PepGMV	ssDNA	Replicase (*AC1*), intergenic region (IR) and coat protein (*AV1*)	hpRNA		[114]
Tomato	potato spindle tuber viroid	PSTVd	(+) ssRNA	Viral sequence	hpRNA		[115]
	cucumber mosaic virus	CMV	(+) ssRNA	*2a* and *2b* genes 3′ UTR	amiRNA	Precursor miR159a	[116]
	cucumber mosaic virus	CMV	(+) ssRNA	Replicase	hpRNA	1138 bp	[117]
	tomato yellow leaf curl virus	TYLCV	ssDNA	Replicase (*C1*)	hpRNA		[118]
	tomato yellow leaf curl virus	TYLCV	ssDNA	Coat protein	hpRNA		[119]
Potato	potato spindle tuber viroid	PSTVd	(+) ssRNA	Ribunuclease *pac*1	dsRNA		[120]
	potato virus X	PVX	(+) ssRNA	ORF2 of PVX	hpRNA	300 bo (ORF2-PVX)	[89]
	potato virus Y	PVY	(+) ssRNA	Protease gene PVY		365 bp (HC-Pro-PVY)	
	potato leaf roll virus	PLRV	(+) ssRNA	CP gene PLRV		300 bp (CP-PLRV)	
	sweet potato chlorotic stunt virus	SPCSV	(+) ssRNA	Replicase	hpRNA		[121]
	sweet potato feathery mottle virus	SPFMV	(+) ssRNA				
	potato virus X	PVX	(+) ssRNA	Coat protein	hpRNA	600 bp: 180 bp (PVX), 240 bp (PVY), 180 bp (PVS)	[122]
	potato virus Y	PVY	(+) ssRNA				
	potato virus S	PVS	(+) ssRNA				
	potato virus Y	PVY	(+) ssRNA	CP gene PVY	hpRNA	600 bp: 200 bp for each target	[102]
	potato virus A	PVA	(+) ssRNA	Cylindrical inclusion body PVA			
	potato leaf roll virus	PLRV	(+) ssRNA	CP gene PLRV			
	potato virus Y	PVY	(+) ssRNA	Coat protein	hpRNA	605 bp	[123]
***Cucurbitacea***	**Virus**		**Type**	**Target gene**	**RNA Type**	**Construct Length**	**Reference**
Melon	zucchini yellow mosaic virus	ZYMV	(+) ssRNA	Coat protein	antisense		[124]
	papaya ringspot virus type W	PRSV-W	(+) ssRNA	Coat protein	hpRNA		[125]
	zucchini yellow mosaic virus cucumber vein yellowing virus	ZYMV CVYV	(+) ssRNA	*Cm*-*eIF4E* translation initiation factors (eIF)	hpRNA (pHANNIBAL)	175 bp	[90]
	melon necrotic spot virus	NSV	(+) ssRNA				
	Moroccan watermelon mosaic virus	MWMV	(+) ssRNA				
			(+) ssRNA				
Cucumber and Melon	zucchini yellow mosaic virus	ZYMV	(+) ssRNA	VSR HC-Pro	hpRNA (pHANNIBAL)	657 bp	[126]
Watermelon	watermelon silver mottle virus	WSMoV	(+) ssRNA	Partial N gene of WSMoV fused to partial CP gene sequences of CMV, CGMMV, WMV	silencer DNA (the middle half N gene of (WSMoV))	449 bp CP-CMV	[127]
	cucumber mosaic virus	CMV	(+) ssRNA			449 bp CP-CGMMV	
	cucumber green mottle mosaic virus	CGMMV	(+) ssRNA			449 bp CP-WMV	
	watermelon mosaic virus	WMV	(+) ssRNA				
***Fabaceae***	**Virus**		**Type**	**Target gene**	**RNA type**	**Construct length**	**Reference**
Soybean	soybean dwarf virus	SbDV	(+) ssRNA	Coat protein	hpRNA	602 bp	[128]
	alfalfa mosaic virus	AMV	(+) ssRNA	highly conserved sequences of one virus	Short dsRNA stems	109 bp (AMV)	[129]
	bean pod mottle virus	BPMV	(+) ssRNA			147 bp (BPMV)	
	soybean mosaic virus	SMV	(+) ssRNA			123 bp (SMV)	
	soybean mosaic virus	SMV	(+) ssRNA	Coat protein	hpRNA		[130]
	soybean mosaic virus	SMV	(+) ssRNA	VSR HC-Pro	hpRNA		[131]
	soybean mosaic virus	SMV	(+) ssRNA	VSR HC-Pro	hpRNA	268 bp	[132]
	soybean mosaic virus	SMV	(+) ssRNA	*P3* cistron	hpRNA	302 bp	[133]
Common bean	bean golden mosaic virus	BGMV	(+) ssRNA	Replicase (*AC1*)	hpRNA	411 bp	[134]
Cowpea	cowpea severe mosaic virus	CPSMV	(+) ssRNA	proteinase cofactor (CPSMV) Coat protein (CABMV)	hpRNA	899 bp (415 bp RNA1- CPSMV and 462 bp CP-CABMV)	[135]
	cowpea aphid-borne mosaic virus	CABMV	(+) ssRNA				
White clover	white clover mosaic virus	WCMV	(+) ssRNA	Replicase	sense, antisense and hpRNA	790 bp	[136]
***Poaceae***	**Virus**		**Type**	**Target gene**	**RNA type**	**Construct length**	**Reference**
Rice	rice tungro bacilliform virus	RTBV	dsDNA	ORF IV	dsRNA	1326 bp	[137]
	rice dwarf virus	RDV	dsRNA	non-structural protein Pns12 and Pns4	hpRNA	500 bp: Pns12-12N	[138]
						499 bp: Pns12-12C	
						500 bp: Pns4-4N	
						504 bp: Pns4-4M	
	rice stripe virus	RSV	(−) ssRNA	Coat protein and special-disease protein (SP)	hpRNA	300 bp (SP); 450 bp (CP)	[139]
						700 bp (SP/CP; CP/SP)	
	rice stripe virus	RSV	(−) ssRNA	Nucleocapsid (pC3), MP (pC4), glycoprotein (pC2), non-structural protein (p4)	hpRNA	500 bp (for each target)	[140]
	rice black streaked dwarf virus	RBSDV	dsRNA	nonstructural Pns9 protein	hpRNA	500 bp	[141]
	rice grassy stunt virus	RGSV	(−) ssRNA	nucelocapsid protein pC5; movement protein pC6	dsRNA	500 bp (for each target)	[142]
Wheat	wheat streak mosaic virus	WSMV	(−) ssRNA	nuclear inclusion protein ‘a’ (*NIa*) gene	hpRNA (pSTARGATE)	696 bp	[143]
	wheat streak mosaic virus	WSMV	(−) ssRNA	Conserved region of WSMV genome	amiRNA	Precursor miR395	[144]
Maize	maize dwarf mosaic virus	MDMV	(+) ssRNA	Coat protein	hpRNA	404 bp	[145]
Barley	barley yellow dwarf virus	BYDV	(+) ssRNA	Polymerase gene	hpRNA	1600 bp	[146]
***Euphorbiaceae***	**Virus**		**Type**	**Target gene**	**RNA type**	**Construct length**	**Reference**
Cassava	African cassava mosaic virus	ACMV	ssDNA	Rep (*AC1*); TrAP (*AC2*), REn (*AC3*)	antisense construct	Full-length of each target gene	[147]
	African cassava mosaic virus	ACMV	ssDNA	bidirectional promoter of ACMV DNA-A	intron-containing dsRNA	256 bp	[148]
	African cassava mosaic virus	ACMV	ssDNA	Rep (*AC1*)	hpRNA	154 bp	[149]
	Sir Lankan cassava mosaic virus	(SLCMV)	ssDNA	AV1 and AV2	hpRNA	527 bp	[117]
	cassava brown streak Uganda virus	CBSUV CBSV	(+) ssRNA	Coat protein	hpRNA	p5001 construct	[150]
	cassava brown streak virus		(+) ssRNA				
	cassava brown streak Uganda virus	CBSUV CBSV	(+) ssRNA	Coat protein	hpRNA	Field trials (Chauhan et al. 2015)	[151]
	cassava brown streak virus		(+) ssRNA				
	cassava brown streak Uganda virus	CBSUV CBSV	(+) ssRNA	Coat protein	hpRNA	525 bp	[152]
	cassava brown streak virus		(+) ssRNA				
	cassava brown streak Uganda virus	CBSUV CBSV	(+) ssRNA	Coat protein	hpRNA	894 bp (full-length CP)	[153]
	cassava brown streak virus		(+) ssRNA			401 bp (CP-NT)	
	cassava brown streak Uganda virus	CBSUV CBSV	(+) ssRNA	Coat protein	hpRNA	Field trials (Yadav et al. 2011)	[154]
	cassava brown streak virus		(+) ssRNA				
	cassava brown streak Uganda virus	CBSUV CBSV	(+) ssRNA	Coat protein	hpRNA	Field trials (Yadav et al. 2011)	[155]
	cassava brown streak virus		(+) ssRNA				
	cassava brown streak virus	CBSV	(+) ssRNA	Coat protein	hpRNA	895 bp CP-CBSV	[156]
	cassava brown streak Uganda virus	CBSUV				898 bp CP-CBSUV	
Poinsettia	poinsettia mosaic virus	PnMV	(+) ssRNA	Coat protein and RNA-dependent RNA-polymerase	hpRNA	500 bp each target	[157]
***Tropical fruits***	**Virus**		**Type**	**Target gene**	**RNA type**	**Construct length**	**Reference**
*Citrus macrophylla*	citrus tristeza virus	CTV	(+) ssRNA	3′ p23 (VSR) and 3′ UTR	hpRNA	900 bp	[109]
Mexican lime	citrus tristeza virus	CTV	(+) ssRNA	3′ p23 (VSR) and 3′ UTR	sense, antisense and hpRNA	549 bp	[158]
	citrus tristeza virus	CTV	(+) ssRNA	VSR p20, p23, p25	hpRNA	548 bp (p20); 629 bp (p23); 670 bp (p25)	[159]
	citrus psorosis virus	CPsV	(−) ssRNA	Coat protein and 54K	hpRNA	372 bp CP; 436 bp 54K	[160]
Sweet orange	citrus psorosis virus	CPsV	(−) ssRNA	Coat protein, 54K or 24K genes	hpRNA	372 bp CP; 436 bp 54K; 312 bp 24K	[108]
Banana	banana bunchy top virus	BBTV	ssDNA	Replicase	hpRNA	CP-full-length; 651 bp partial CP sequence	[92]
***Brassicaceae***	**Virus**		**Type**	**Target gene**	**RNA type**	**Construct length**	**Reference**
Arabidopsis	turnip yellow mosaic virus	TYMV	(+) ssRNA	VSR P69 (TYMV)	amiRNAs	Precursor miR159a	[161]
	turnip mosaic virus	TuMV	(+) ssRNA	VSR HC-Pro (TuMV)			
	cucumber mosaic virus	CMV	(+) ssRNA	3′ UTR	amiRNAs	Precursor miR159a	[162]
***Others***	**Virus species**		**Type**	**Target gene**	**RNA type**	**Construct length**	**Reference**
*Asteraceae*	chrysanthemum virus B	CVB	(+) ssRNA	Coat protein	sense, antisense and hpRNA	273 bp	[163]
*Amaranthaceae*	beet necrotic yellow vein virus	BNYVV	(+) ssRNA	Replicase	hpRNA	459 bp, 589 bp and 824 bp	[164]
*Andropogoneae*	sugarcane mosaic virus	SCMV	(+) ssRNA	Coat protein	hpRNA	Not mentioned	[165]
	sorghum mosaic virus	SrMV	(+) ssRNA				
*Vitaceae*	grapevine fanleaf virus	GFLV	(+) ssRNA	Coat protein	amiRNA	Precursor miR319a	[91]
